# Land finance, infrastructure investment and housing prices in China

**DOI:** 10.1371/journal.pone.0292259

**Published:** 2023-10-24

**Authors:** Mengkai Chen, Ting Chen

**Affiliations:** 1 School of Management Science and Engineering, Anhui University of Technology, Ma’anshan, China; 2 Key Laboratory of Multidisciplinary Management and Control of Complex Systems of Anhui Higher Education Institutes, Anhui University of Technology, Ma’anshan, China; Shenzhen University, CHINA

## Abstract

Housing prices in China have experienced rapid growth in recent decades, and land finance has long been discussed as an important factor in this growth. In this paper, we explore the interactions among housing prices, land transfer revenue and infrastructure investment from the perspective of government revenue and expenditure. Based on the panel data of 35 large and medium-sized cities in China from 2000 to 2017, the empirical results show that land transfer revenue, infrastructure investment and housing prices are causally related and result in positive feedback. The grouped regression results show that infrastructure investment has greater impacts on housing prices in eastern region cities than in the other cities. In contrast, in the central and western regions, land sales revenue has a greater impact on housing prices, indicating that cities in less-developed areas are more dependent on land finance than are those in more developed regions. Finally, we use the vector error correction model (VECM) to add control variables for robustness testing. The results show that land transfer income and infrastructure investment have a positive impact on housing prices. Our results provide some references for the stable development of housing markets in China.

## 1. Introduction

In recent decades, many economies around the world have experienced a rapid growth in housing prices. The risk of an economic bubble triggered by rising housing prices has fostered widespread concerns and further threated macroeconomic development, especially in rapidly developing countries [1]. Especially in China, the housing market has experienced an unprecedented boom since the housing reform [2]. According to the National Bureau of Statistics, the average price of commercial housing (Since the real estate market reform in the 1980s, China’s housings consisted of government-provided affordable housing and market-provided commercial housing. Most affordable housing is owned by the government and cannot be traded through the market. Commercial housing refers to housing developed by development enterprises by purchasing land, the property rights of which can be traded through the market. We focus on commercial housing prices in this study.) rose from 2,117 yuan/m^2^ to 10,396 yuan/m^2^ from 2001 to 2021, respectively, an increase of more than 5 times during the past two decades. According to Li et al. [3], the repayment-to-income ratio of the top 1% cities in China reached 1.16 in 2018, which is higher than that of other cities around the world, such as New York, London and Hong Kong. The rapid rising housing prices have put pressure on housing affordability for the people in China. Therefore, the Chinese government has explicitly listed “meeting the housing needs of all people” as one of its top goals in the policy agenda.

In addition to macroeconomic development [4, 5], monetary policy [6],wages and employment [7], interest rates [8], and inflation (Consumer Price Index(CPI)) [9, 10], China’s unique land policy has long been discussed as an important factor influencing housing prices. The tax-sharing reform implemented in China in 1994 adjusted the financial distribution between the central government and local governments [11]. This adjustment enabled local governments to more flexibility develop economically but also brought with it high financial pressure since the central government retained control over fiscal revenue. Since local governments possess land use rights, the selling of land use rights has been widely adopted by local governments to enhance investment and fiscal revenue, especially given that the assessment of local governments is highly reliant on economic development [12]. Such widespread adoption has resulted in the high reliance of local governments on urban land-related finance, which is often called land finance (*tu di cai zheng* in Chinese). In recent decades, more than 40% of local government fiscal revenue has been derived from land finance. Since land values are highly related to the housing market, local governments are more inclined to sell land during real estate booms than during other periods [13]. In contrast, the recent cooling down in China’s housing and land market has resulted in great financial pressure being placed on local governments, and thus, local governments have introduced a series of loose regulatory policies to stimulate enterprises to buy land and invest.

In addition to the causality of land finance, we suggest that infrastructure investment is also a driving factor of housing prices. In fact, as the main content of local fiscal expenditure, infrastructure investment is not only an efficient way in which to increase GDP but also an important measure through which to improve the living environment and urban attractiveness. In 2021, more than 50% of China’s investments were in infrastructure. Since land use rights and the land supply structure are dominated by local governments, whether they are more inclined to increase the level of infrastructure investment by raising land revenue remains unclear. Several studies have analyzed infrastructure as a prerequisite for obtaining finance for land sale [14]. Some studies suggest that local governments can reap the rewards of land finance [15]. Although a large body of literature has empirically examined the direct or indirect role of infrastructure in promoting local economies [16], the causal relationships among land finance, infrastructure investment and housing prices are still unclear.

Based on the panel data of 35 large and medium-sized cities in China from 2000 to 2017, this study reveals that both land transfer revenue and infrastructure investment have a positive impact on housing prices and, inversely, that the rise in housing prices also promotes land transfer revenue and infrastructure investment, forming a positive feedback cause and effect. Furthermore, our heterogeneity results suggest that infrastructure investment has greater impacts on housing prices in eastern cities than in other cities. In contrast, in the central and western regions, land transfer revenue has a greater impact on housing prices, indicating that cities in less-developed areas are more dependent on land finance. Finally, we use the vector error correction model (VECM) and add control variables to conduct a robustness test. The results show that when the housing price deviates from the equilibrium state in the short term, the error correction term exerts a reverse correction mechanism on the housing price and has a certain self-adjustment ability.

The contributions of this study can be highlighted as follows. First, this study enriches relevant research in the field of land finance, where the relationship between land policies and housing prices has been widely explored [7, 8, 17, 18]. This paper constructs a research framework including land finance, infrastructure investment and housing prices, discusses the factors influencing housing price increases from the aspects of land fiscal expenditure and income, and provides some new perspectives on the growth of literature on China’s real estate market. Second, in contrast the study of Chen, Long and Qin [19], we study the regional heterogeneity of the relationship between land finance, infrastructure investment, and housing prices. Third, our results suggest that local governments should change their excessive reliance on land finance for economic development and broaden their finance channels for infrastructure investment, which provides a reference from which the Chinese government can implement urban policies.

The rest of the paper is arranged as follows. Section 2 introduces the background of China’s land transfer system and reviews the related literature. Section 3 describes the empirical design and data selection. Section 4 reports the empirical results. Section 5 analyzes the heterogeneity test. Section 6 carries out the robustness test, Section 7 presents the conclusions and policy implications.

## 2. Literature review

### 2.1 Background of the land transfer system in China

After the foundation of the People’s Republic of China in 1949, all land was monopolized by the state or collectives. Companies, organizations and individuals could obtain nontransferable land use rights from the Chinese government through non-market-oriented land distribution. During this stage, the government completely monopolized the land market and dominated the amount of land and the timing of development [20]. However, this highly centralized way of planning and allocating resources also had some drawbacks. In cities, the uncompensated and indefinite allocation model of state-owned construction land made land use inefficient. In rural areas, the presence of centralized operation and concentrated labor reduced farmers’ enthusiasm for production and agricultural production efficiency. In addition, the control method of ownership and use rights presented shortcomings in coordinating the interests of relevant subjects.

After the reform and opening in the 1980s, the central government gradually reduced its direct intervention and control over the economy and society and allowed some elements of society to flow freely, providing society with more vitality through decentralization [21]. The land system underwent a series of adjustments, such as moderately relaxing the control of urban and rural land use rights, separating land ownership and use rights, and promoting the circulation of use rights. The separation of land ownership and use rights mobilized farmers’ enthusiasm for production, agricultural productivity was greatly improved, and the increase in the degree of power of local governments formed its independent interest model. A remarkable sign was the entry into force of Article 10 of the Constitutional Amendment on April 12, 1988, which stipulated the possibility of transferring land use rights. Based on this amendment, land use rights were separated from land ownership, and there were two main ways in which to obtain land use rights. Companies, organizations, and individuals (including foreigners) could negotiate land use rights with governments an agreement that incorporated a substantial granting fee that was paid in full and upfront. This granting fee was based on publications from China’s Ministry of Land and Resources, which stated minimum pricing standards for grants, as predetermined based on localities throughout the country. Alternatively, land demanders could purchase land use rights in the secondary land market from existing rights holders. In this case, both buyers and sellers, who were typically state-owned enterprises, negotiated a price for the land under consideration.

Under this negotiation system, it was convenient for Chinese state-owned or collectively owned land developers to negotiate land lease agreements with either municipal governments or other state-owned enterprises. However, non-state-owned land developers, especially foreign developers, faced serious difficulties in accessing the Chinese real estate market, mainly due to barriers to communication with the government. Since then, the land reserve system has not yet been fully established. Land rights traded on the market during the period 1988–2002 remained very high. Most of the demand for land could be met in the secondary land market. The problem with pre-2002 land transfer policies was that buyers and sellers could report contract prices that were much lower than actual transaction prices to reduce the amount of transaction fees paid to the government, which caused significant losses in government revenue [22]. To promote transparency in the land market, China’s Ministry of Land and Resources began to implement a numerous new policies on granting state-owned land use rights on July 1, 2002. Through these policies, land developers could enter the Chinese housing market by obtaining land use rights through fair competition in the market. As a result, these policies inherently made markets more transparent.

### 2.2 Local government land finance behavior

**Fig 1:**
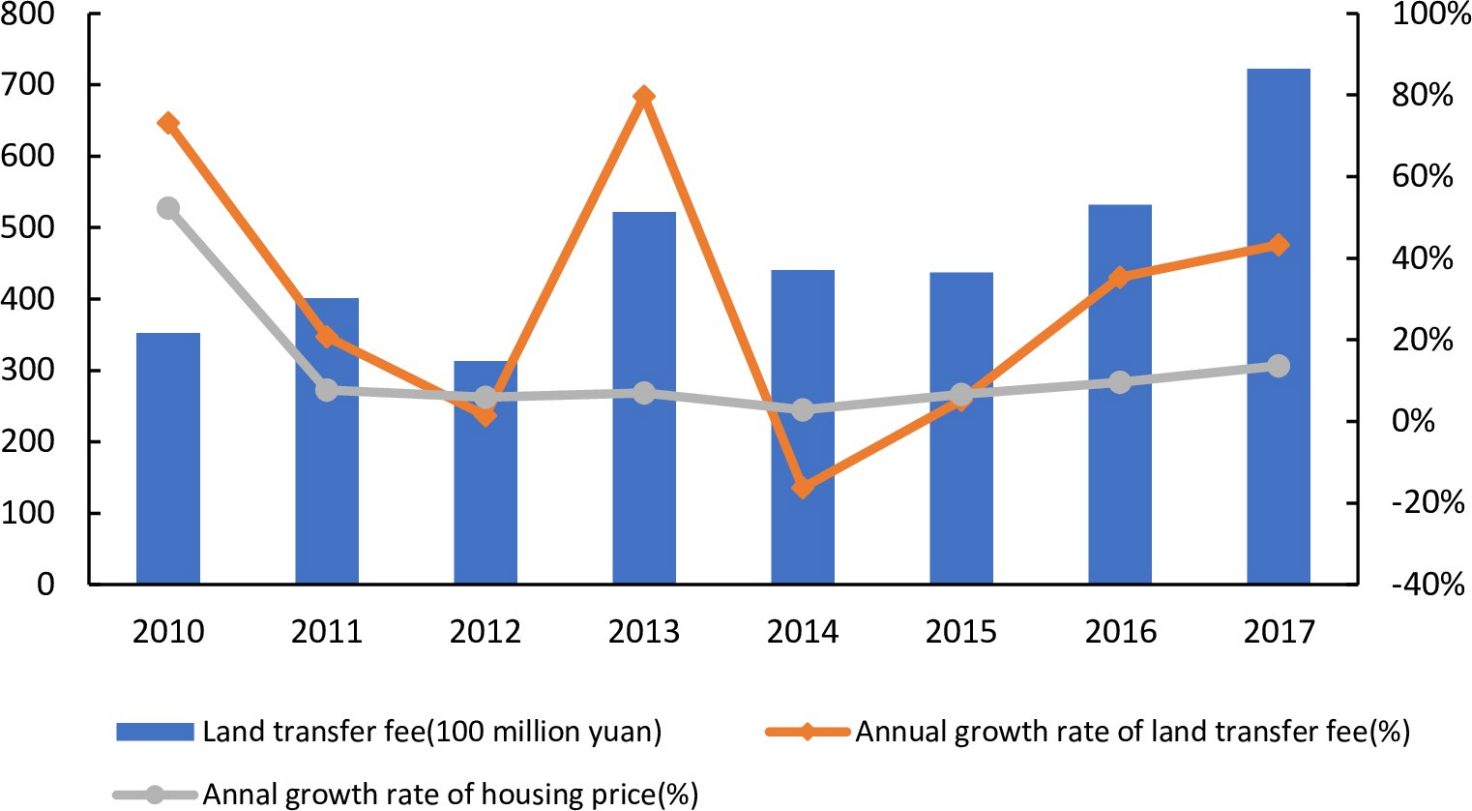
Land transfer fee and housing prices.

After the reform of the tax-sharing system in 1994, the Chinese government’s de-centralization system underwent changes in terms of decentralization and financial collection. The "fiscal pressure" brought about by the reform is the main reason for land finance [23], and local governments have chosen to use the increasing "land value" to generate revenue to fill the fiscal gap. In 2004, the "bidding, auctioning and listing" system with "the highest price wins" motto at its core began to be implemented, marking an important turning point in the composition of China’s real estate market. Given the rapid level of urbanization in recent years, this bidding strategy has resulted in increasing residential land values [24, 25]. Moreover, to develop the economy and to provide more employment opportunities, local governments often sell lands labelled "industrial" to enterprises at a lower price through agreements [24]. This situation could also increase the demand for housing and thus lead to an increase in housing prices [26]. In 2006, China’s land transfer revenue was 0.7 trillion yuan, and in 2017, this income exceeded 1.2 trillion yuan. Over the next 10 years, land sale income continued to rise. Therefore, this land policy is considered the main factor in the rapid boom in supply-side housing prices [27]. For example, Du [28] found the existence of a long-term equilibrium between urban land and housing markets in China, with the rise in land prices being the main cause of rising housing prices in the short run. From the opposite perspective, the uncertainty of housing prices may delay development times and increase land prices [29]. As shown in Fig 1, the growth rates of land transfer income and housing prices in recent years are basically the same. Especially after 2014, both land transfer revenue and housing prices have experienced substantial growth.

Regarding the relationship between land finance and housing prices, scholars have three main views. The first view posits that housing prices cause a rise in and are the Granger cause of land prices [30, 31]. From the point of view of demand, the price of land products determines the rent. Coats and Ricardo [32] suggested that the price of land was determined by the value of those goods produced on it, While Beesley and Alonso [33] suggested that the highest bid would naturally be chosen when choosing different uses and, thus, that rising housing prices would lead to an increase in the price of land plots in the corresponding area and an overall increase in land prices. The second view posits that the rise in land prices is the reason for the rise in housing prices [34]. Land in most Western countries is privately owned, and land trading markets are more open and competitive than is that in China. The theory that land prices promote the housing price considers mainly that land prices are the basis of real estate price, suggesting that land prices are the main component of housing prices and that an increase in land prices [35]. The third view posits that there is a complex mutual influence relationship between housing prices and land prices [36, 37]. On the one hand, the boom in the real estate market led to higher land prices, and local governments became more willing to increase the land supply to increase their fiscal revenue. On the other hand, excess land finance is one of the reasons for rising housing prices. Wang and Hou [38] found that land finance accounts for 39.01% of housing prices, which is much higher than other factors such as monetary easing policy, labor preference and housing demand shocks. Moreover, Jiang and Qiu [39] argued that the land purchase price has a greater positive impact on the average selling price of residential commercial housing when considering residents’ disposable income. Therefore, scholars believe that housing prices and land prices do not present a one-way influence relationship [40, 41]. Housing prices and land prices interact in a dynamic process that needs to be analyzed from a more comprehensive perspective.

### 2.3 Infrastructure investment

Since the 1994 tax-sharing reform and the beginning of urbanization, local governments have become more dependent on land transfers to finance public spending, especially infrastructure investment. Early studies in other countries considered the need for local governments to invest in public goods (infrastructure). For example, Tiebout [42], in his paper "A Pure Theory of Local Expenditure", proposed that the general equilibrium solution of local governments providing public goods is optimal. Further expansions of the model by Hamilton [43] and Fischel [44] concluded that the quality and cost of local public goods affect the value of local housing. In China, since land is state owned, the government can exploit the monopoly rent of land and generate high taxes [45]. The further expansion of land finance makes it a major contributor to infrastructure investment [6]. As a result, land revenue plays an increasingly important role in local expenditures. Some studies have noted that land finance can be used to improve or upgrade infrastructure [15]. Relatively little research has focused on the link between land finance and infrastructure development and how land finance can finance urban infrastructure development [46]. Both developed and developing countries use land to invest in infrastructure [47]. Land sale revenue is the main source of financing for urban infrastructure development. Previous empirical studies have shown that land finance can improve the fiscal capacity of local governments, especially for the development and improvement of urban infrastructure in China [24]. Due to the fact that the central government’s assessment of local officials mainly de-pends on GDP, local officials have a strong incentive to rely too much on land transfer for infrastructure investment development to enhance their performance [48].

**Fig 2:**
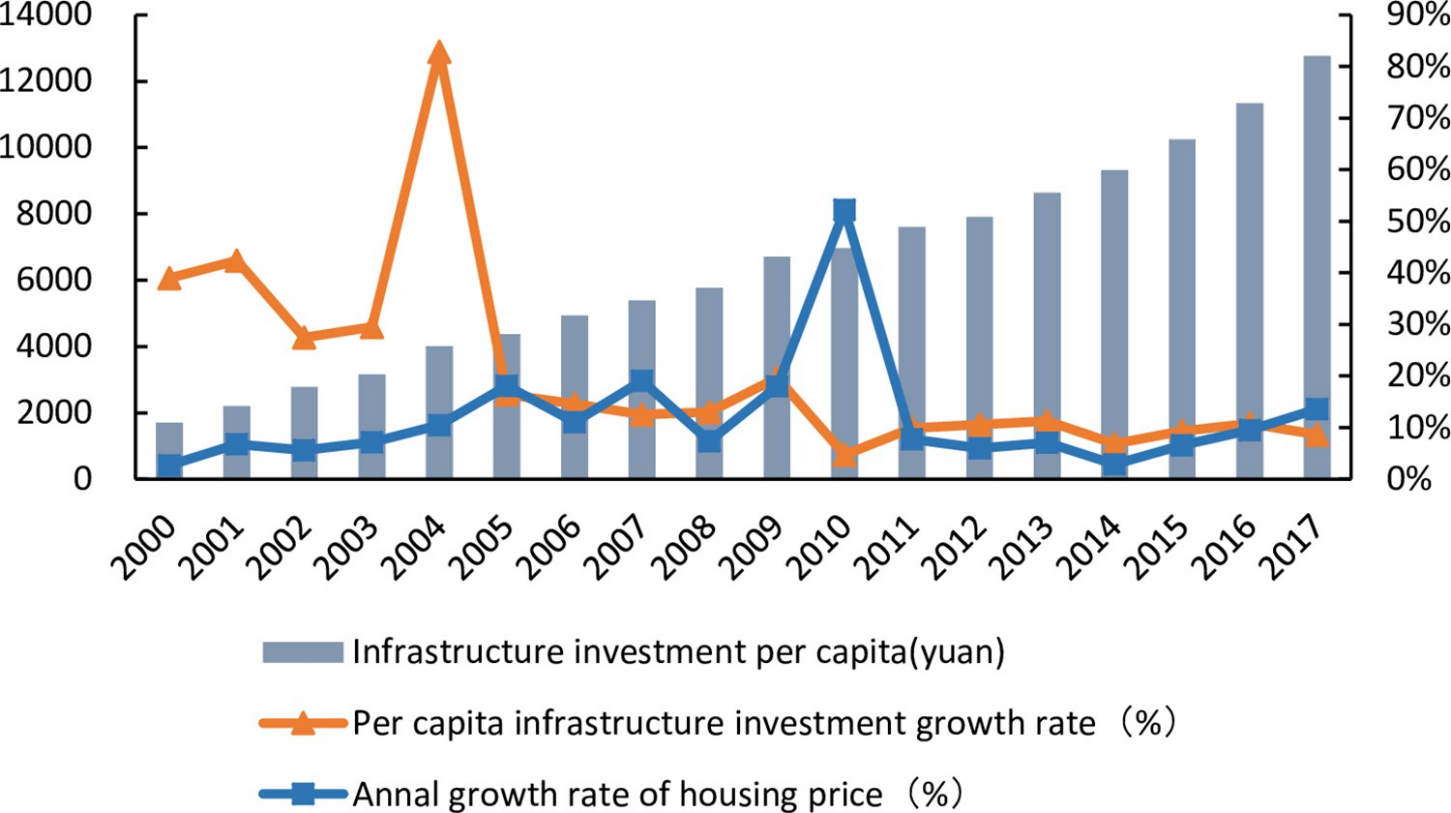
Infrastructure investment and housing prices.

As a special commodity, housing prices are affected by the infrastructure of surrounding cities. As the carrier and support system of urban economic development and urban construction, urban infrastructure investment plays an important role in promoting the rapid development of the local economy. Therefore, local governments have a strong incentive to expand urban infrastructure investment [49].The increase in the level of urban infrastructure investment improves urban public services. The influx of people from other cities has increased the demand for housing. Similarly, the increase in the level of urban infrastructure investment has developed the urban economy, increased per capita income, increased the ability to buy housing, and has eventually been reflected in the rise in housing prices. Therefore, the increase in the level of urban infrastructure investment has contributed to the rise in housing prices to some extent. Fig 2 shows the relationship between the growth rates of per capita infrastructure investment and housing prices. With the continuous growth of the level of per capita infrastructure investment, housing prices also show the same fluctuating trend.

Previous research has demonstrated that social infrastructure earns high price premiums in China’s urban housing markets. One important channel is that infrastructure investment could create a positive externality in the housing market by improving the amenities and accessibility of districts [50]. One of the most prominent example is access to prestigious schools, as nearby schools can influence housing prices. Specifically, the aspirations of wealthy Chinese parents to provide their children with high-quality education increase their willingness to pay higher housing costs [51]. The presence of high-quality schools in an area can lead to an increase in district housing prices of 14%, a phenomenon prevalent in urban China [16]. Accessibility to hospitals also influences both housing prices and rents [52]. However, although hospitals are not usually considered and infrequently studied regarding capitalization, proximity to regions with good quality hospitals positively influences housing prices in those regions [53]. These findings indicate different scenarios in urban and rural China with respect to the capitalization of hospital facilities. Finally, accessibility to recreational facilities and an increasing focus on health and wellness play a key role in determining housing preferences [54]. A considerable body of research suggests that the presence of recreational facilities (e.g., sport stadiums and public parks) positively influences residential property values [55].

The above literature review shows that the relationship between land finance and housing prices has been widely discussed. Moreover, the impact of the level of local infrastructure investment on housing prices has also attracted widespread attention. However, few studies have directly investigated the factors influencing housing prices from the perspective of government income and expenditure. In addition, whether these factors vary across different regions is still remain understanding. In this paper, we innovatively explore the interactions among land finance, infrastructure investment and housing prices, and discuss the potential heterogeneity among them, which not only enriches the literature but also provides some references for China’s housing reform.

## 3. Data description and research design

### 3.1 Data description

In this study, we construct panel data on 35 large and medium-sized cities from 2000 to 2017 (The data of land transfer income is only available until 2017). All data are obtained from the China Land and Resources Statistical Yearbook, China Real Estate Statistical Yearbook and China Statistical Yearbook.

The dependent variable is housing price (HP), which is the sales volume of commercial housing divided by the sales area. We adopt land transfer income as a proxy variable for land finance (LF). Infrastructure expenditure (IE) is represented by the per capita amount of infrastructure investment. To mitigate heteroscedasticity, all variables are treated logarithmically. The descriptive statistics are shown in Table 1.

**Table 1:** Descriptive statistics.

Variables	Obs.	Mean	Min.	Max.	Std. Dev.
HP	630	8.4760	7.1940	10.7780	0.6810
LF	630	13.3640	7.0830	17.1180	1.9730
IE	630	8.4840	4.8890	10.3020	0.8340

### 3.2 Research design

The main purpose of this paper is to investigate the fluctuation in housing prices across the country and across regions. This paper uses panel data to establish an in-variant parameter model. The long-term equilibrium equation regarding real estate prices is as follows:

$${HP}_{i,t}=\alpha_{1}+\alpha_{2}{LF}_{i,t}+a_{3}{IE}_{it}+\mu_{it}$$ (1)

where *a*_1_ is the constant term of the model, *a*_2_ and *a*_3_ are the coefficients of the explanatory variables and *μ*_*it*_ is the random error term. *i* and *t* represent the city and year, respectively. By estimating model (1), the error correction model (ECM) can be obtained by slight deformation and replacement:

$$\Delta {HP}_{it}=\beta_{1}+\beta_{2}{\Delta LF}_{i,t}+\beta_{3}\Delta {IE}_{it}+\beta_{4}\Delta {HP}_{it-1}-\lambda {ecm}_{it-1}+\varepsilon_{it}$$ (2)

where *β*_1_ is the constant term of the model, *β*_2_, *β*_3_, and *β*_4_ are the explanatory variable coefficients, *ε*_*it*_ denotes random error terms, and *ecm*_*it*−1_ is the error correction term, which reflects the degree of housing price deviation from the equilibrium state.

To test heterogeneity, we divide the panel data of 35 large and medium-sized cities into eastern, central and western cities, to better reflect the impact of land transfer income and infrastructure investment on housing prices in different regions.

## 4. Empirical testing steps and analysis

### 4.1 Stability results

To prevent the emergence of "pseudo regression", we adopt the LLC method developed by Levin, Lin and Chu [56] and the ADF method developed by Dickey and Fuller [57]. The ADF is used to test for root units containing higher-order sequence correlations. The Breitung [58] and Phillips and Perron (PP) [59] methods are used to test the stationarity of each variable. The results are shown in Table 2. The P values of the three variables pass the LLC and PP tests. However, the remaining variables are not significant. All of them cannot reject the null hypothesis, and the test result is greater than 0.05.

**Table 2:** Original sequence results.

Methods	HP		LF		IE	
	T	P	T	P	T	P
ADF	5.5091	1.0000	1.5904	0.9441	-0.4429	0.3289
LLC	-2.2425	0.0125	-4.3156	0.0000	-4.6347	0.0000
Breitung	-1.9414	0.0261	-1.9177	0.0276	1.2480	0.8697
PP	6.1868	1.0000	-1.6504	0.0494	-2.7759	0.0028

We then carry out the first-order differential for all variables and retest the stationarity, and the results are shown in Table 3. The P values of all variables under the four testing methods are significant at the 1% level, indicating that the three variables are stationary after first-order differential processing and that these three variables present first-order stationarity.

**Table 3:** Results of first-order differential sequences.

Methods	HP		LF		IE	
	T	P	T	P	T	P
ADF	5.5091	1.0000	1.5904	0.9441	-0.4429	0.3289
LLC	-2.2425	0.0125	-4.3156	0.0000	-4.6347	0.0000
Breitung	-1.9414	0.0261	-1.9177	0.0276	1.2480	0.8697
PP	6.1868	1.0000	-1.6504	0.0494	-2.7759	0.0028

### 4.2 Cointegration test

According to the previous analysis, Δ*HP*, Δ*LF*, and Δ*IE* are all first-order monointeger variables, which can be considered cointegrated tests. To ensure the reliability of the conclusions, a panel cointegration test was performed using the Pedroni statistic and Kao’s ADF statistic. The test results are shown in the following table. The Pedroni method constructs multiple statistics for the panel cointegration test based on regression residuals. Except for Panel V with the right-tailed test, the remaining statistical tests are all left-tailed tests, three of which use the joint intragroup scale description, namely, Panels V, PP and ADF. The other three tests are described using between-group scales. If all statistics reject the null hypothesis that there is no cointegration at a significant level, then there is a cointegration relationship between nonstationary sequences. From the test results in Table 4, except for Spearman’s rho statistic, the P values of the other statistics are less than 0.05. Consider a panel cointegration relationship between HP, LF, and IE. The null hypothesis is rejected, and there is a tripartite panel cointegration relationship among HP, LF and IE. This satisfies the establishment of Granger’s causality test and the panel ECM.

**Table 4:** Panel cointegration test results.

Method	Hypothesis	Statistics	t-Statistic	P
Kao	*H*_0_: ρ = 1	ADF	-3.0228	0.0013
		Panel v Statistic	1.9696	0.0244
		Panel rho Statistic	-0.1250	0.4503
	*H*_0_: ρ = 1	Panel PP Statistic	-3.1170	0.0009
Pedroni	*H*_1_:	Panel ADF Statistic	-3.9927	0.0000
	(*ρ*_*i*_ = ρ) <1	Group rho Statistic	2.3027	0.9894
		Group PP Statistic	-2.7027	0.0034
		Group ADF Statistic	-4.6514	0.0000

### 4.3 Stability test

**Fig 3:**
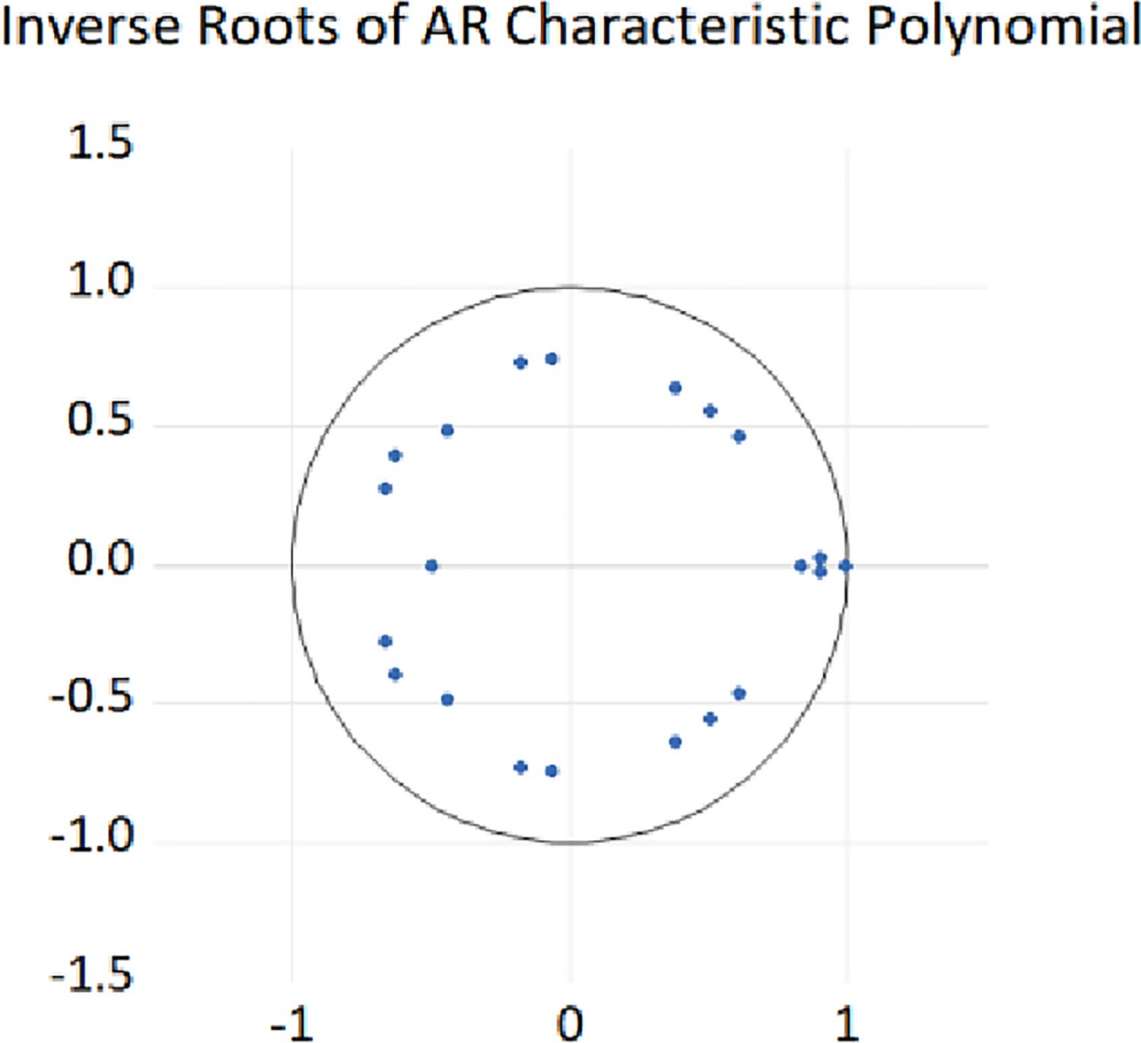
The distribution of AR roots.

We used time series data from HP, IE, and LF to build a vector autoregressive (VAR) model to determine whether a long-term tripartite relationship exists among the three. The results are shown in Fig 3. All the unit roots of the above variables are located in the unit root circle, indicating that the VAR model is stable, which shows that such a long-term stable relationship exists.

### 4.4 Causality test

To further examine and verify the causal relationship among HP, LF, and IE, Dumitrescu Hurlin’s (DH’s) [60] extended test is used. The panel causal test of DH allows the regression coefficients for each cross-sectional element to be variable (i.e., the coefficients differ between individuals at the same time). In economics, the significance of DH causality is also meaningful and can still play a role in economic forecasting. In this paper, lag dates of 1 and 2 years are selected, and the results are shown in Table 5. First, housing prices and land transfer income affect each other, and an increase housing prices helps promote the growth of local land transfer income. The increase in land sale income has further pushed up house prices. Second, housing prices and infrastructure investment affect each other, and the increase in the urban population has led to the vigorous development of urban infrastructure construction, thereby pushing up housing prices. Infrastructure is one of the most important factors in rising housing prices, and rising housing prices contribute to infrastructure improvements. Finally, land transfer income and infrastructure investment affect each other, the growth of land transfer income strongly supports the growth of the level of infrastructure investment, and urban infrastructure construction attracts more foreigners while causing land price increases, breaking the balance of land supply exceeding the reduction, and promoting an increase in land transfer income.

**Table 5:** Results of Granger causality test.

Granger Hypothesis	Lag by one period	Lag by two period	Whether to reject the null hypothesis
HP is not the Granger cause of LF	5.4272	1.6216	Yes
LF is not the Granger cause of HP	19.2497	18.8649	Yes
HP is not the Granger cause of IE	5.0745	4.8283	Yes
IE is not the Granger cause of HP	8.5634	6.8792	Yes
LF is not the Granger cause of IE	7.5275	5.6971	Yes
IE is not the Granger cause of LF	2.9953	5.0639	Yes

To further verify the mutual influence of land transfer income, infrastructure investment and housing prices, this paper conducts a simple regression analysis. The results are shown in Table 6, which suggest that the above three variables are mutually influenced. This study also found that the coefficient of land transfer income on housing price 0.235 is larger than that of infrastructure on housing price 0.189, indicating that land finance has a greater impact on housing price. This is mainly because land price, as an important part of housing price, affects the pricing behavior of developers to a certain extent. That is, developers will pass on the cost of land use to buyers by raising the price of housing, thus promoting the rise of housing prices. The coefficient of land transfer income to infrastructure investment 0.875 is much larger than that of infrastructure investment on land transfer income 0.202, indicating that due to the promotion pressure of government officials, local governments are overly dependent on land finance for infrastructure investment to develop the economy.

**Table 6:** Regression analysis.

Variable	HP	LF	IE
HP		0.738***	0.728***
		(0.0242)	(0.0717)
LF	0.235***		0.845***
	(0.00837)		(0.138)
IE	0.189***	0.202***	
	(0.0188)	(0.0640)	
Constant	3.731***	1.323***	8.425***
	(0.130)	(0.377)	(0.529)

Note

***p<0.01.

### 4.5 Error correction model

Based on the above tests, we establish an ECM for housing prices, land transfer prices and infrastructure investment, and the long and short-term results are shown in Table 7.

**Table 7:** Basic test results.

Variable	Coefficient	t-Statistic	Prob.
**Result 1 long-term equilibrium effects**			
*C*	3.5619	22.4956	0.0000
LF	0.1802	24.6328	0.0000
IE	0.2271	10.5816	0.0000
**Result 2 short-term volatility effects**			
*C*	0.1170	13.8121	0.0000
*D*(*LF*)	0.0310	3.7019	0.0000
*D*(*IE*)	-0.0041	-0.2203	0.8271
*D*(*HP*(−1))	0.2915	-7.2017	0.0000
*ecm*(−1)	-0.0643	-3.1500	0.0000

Regarding to the long-term equilibrium equation, from the regression results in Table 7, the goodness-of-fit (*R*^2^) of the model is 0.69, and all coefficients pass the F test. Therefore, the model results are convincing. According to the results, for every 1% increase in land transfer income, housing prices increase by 0.18%, and for every 1% increase in the level of infrastructure investment, house prices rise by 0.23%. The coefficients of the long-term equilibrium equation show that land sale revenue has less of an impact on housing prices than on infrastructure investment. Developed infrastructure can not only increase a city’s economy and attract a high level of investment but also provide an impetus for the city’s subsequent development. Economically developed areas can also attract migrants to work, stimulate housing demand and push up housing prices. Moreover, infrastructure construction requires considerable capital investment, and land finance meets the requirements of local governments.

Regarding the short-term fluctuation equation, the long-term equilibrium model obtained by the above analysis generates error correction terms. The above sequence is a first-order stationary sequence. The goodness-of-fit (*R*^2^) of the model is 0.133, and all coefficients pass the F test. Therefore, we consider a significant short-term linear relationship among housing prices, land sale income and infrastructure investment. From the variable coefficient, the previous housing price has positive and significant impact on the current housing price. The impact coefficient of land transfer income on housing prices is 0.031, indicating that for every 1% increase in land transfer income, housing prices increase by 0.031%. However, the level of infrastructure investment has little impact on housing prices. A 1% rise in the level of infrastructure investment leads to a 0.004% drop in housing prices. The error correction coefficient -0.06, indicates that when the short-term fluctuation deviates from the long-term equilibrium value, the error term is adjusted in the opposite direction with a force of 0.06 to return to the equilibrium state. In the short term, land transfer income has a positive stimulating effect on housing prices. On the one hand, the rising trend of housing prices has laid the financial foundation for the increase in land transfer prices, while on the other hand, the land sale method supported by the "auction listing" system has laid the institutional foundation for the price increase of land transfer. The coefficient of infrastructure investment is not significant. Infrastructure construction is affected by different periods in different cities and different areas of the same city, and thus, its differential impact on housing prices is not significant.

The long-term elasticity coefficient of land transfer income and infrastructure investment on housing prices is much greater than is that of short-term elasticity. It can be inferred that the impact of land transfer income and infrastructure investment on housing prices has a certain time lag, which is due to a certain cycle between real estate construction and the provision of public goods. The land purchased by real estate developers is not directly used for current real estate construction. The land needs to be planned for a period before it can be officially put under construction. Therefore, the cost of land transfer in the current period is not included in the current housing price. Similarly, the construction and improvement in infrastructure and the fiscal expenditure related to people’s livelihood education, security, transportation, etc., takes some time to be reflected in the improvement in the regional environment. This situation is one not enjoyed by current home buyers and that cannot be reflected in the current fluctuations in real estate prices.

**Fig 4:**
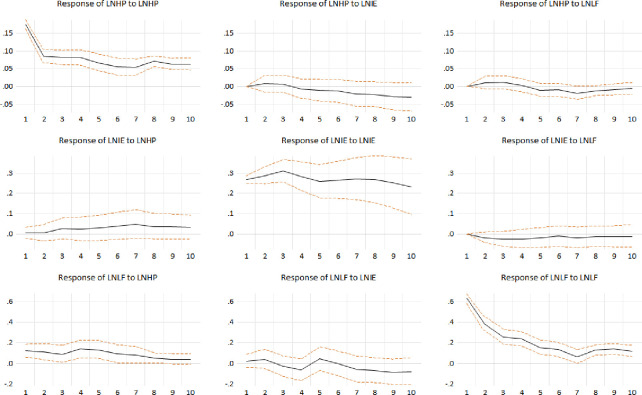
Impulse response diagram.

According to the results of the ECM, we draw an impulse response diagram among the three variables, as shown in Fig 4. The positive impulse response diagram shows the dynamic effect of a variable’s shock on itself or on other variables. As seen from Fig 4, the impact of variables on themselves is always positive and gradually converges to 0, and the trends of all graphs are stable and within the dashed line range of the error term. The impact of IE on HP continues to be positive, and there is no obvious convergence in the 10th phase, indicating that IE has a stable and lasting impact on HP. We focus on the impact response of LF to HP. The impact peaks in the fifth period and then slowly decreases, indicating that LF has a long-term support effect on HP. It can be considered that the excessive transfer of land has a greater positive impact on housing prices.

## 5. Heterogeneity testing

The above empirical analysis focuses on the overall situation across China but ignores regional differences in the impact of land transfer income and infrastructure investment on housing prices. This paper further explores the regional impact of land transfer income and infrastructure investment on housing prices. We divide the sample into eastern and central and western cities and study the heterogeneity of land transfer income, infrastructure investment, and housing prices. The long-term equilibrium results and short-term fluctuation results are shown in Table 8.

**Table 8:** Heterogeneity test results.

Variable	Coefficient		t-Statistic		Prob.	
	Eastern	Central and Western	Eastern	Central and Western	Eastern	Central and Western
**Result 1 long-term equilibrium effects**						
*C*	2.0448	4.3999	5.7123	31.7838	0.0000	00.0000
LF	0.2259	0.1939	13.1420	21.5901	0.0000	00.0000
IE	0.4155	0.1647	8.1751	9.7016	0.0000	0.0000
**Result 2 short-term volatility effects**						
*C*	0.0915	0.1056	7.9606	8.8720	0.0000	0.0000
*D*(*LF*)	0.0396	0.0444	4.2430	2.8586	0.0000	0.0000
*D*(*IE*)	0.0666	-0.0117	1.6732	-0.4245	0.0950	0.6710
*D*(*HP*(−1))	0.0119	-0.3085	0.1841	-5.9610	0.8541	0.0000
*ecm*(−1)	-0.0268	-0.2085	-1.4358	-5.5128	0.1524	0.0000

Form the empirical results of eastern cities, land transfer income and infrastructure investment can be shown to better explain housing price fluctuations. From the long-term results, it can be seen that for every 1% increase in land transfer income, housing prices in eastern cities increase by 0.23%. For every 1% increase in the level of infrastructure investment, housing prices rise by 0.42%. Moreover, it can be seen that land transfer income and infrastructure investment have a significant positive effect on housing prices in eastern cities. From the short-term results, the impact coefficient of infrastructure investment on housing price 0.07 is larger than that of land transfer income 0.03. The error correction coefficient of -0.03 indicates that when short-term fluctuations deviate from the long equilibrium value, the error term is inverted to 0.03 to promote the return to equilibrium. From the long-term and short-term results, housing prices are more affected by infrastructure investment than by land transfer income. This finding is due to the central government’s bias toward land policy in the central and western regions and the overall land shortage in the eastern region, thus, the amount of land transfer income that can be obtained has gradually decreased. The unique geographical location of the eastern region makes its economic development far exceed that of the central and western regions. Therefore, the eastern region has received more investment due to its geographical location. Moreover, to further enhance the overall image of the city, the city’s infrastructure has been vigorously built, thereby increasing housing prices.

Regarding the empirical results of cities in the central and western regions, it is shown that urban land sale income and infrastructure investment are more likely to explain housing price fluctuations. From the long-term results, it can be seen that every 1% increase in land transfer income promotes the increase in housing prices in the central and western regions by 0.19%. For every 1% increase in infrastructure investment, housing prices increase by 0.16%. Land transfer income and infrastructure investment have a significant positive effect on housing prices in central and western cities. The impact of land transfer income on housing prices is greater than that of infrastructure investment. From the short-term results, it can be seen that the land transfer income of the central and western region governments has the greatest impact on housing prices, and a 1% increase in land transfer income promotes a 0.04% increase in housing prices. Infrastructure investment has the weakest impact, with housing prices rising 1% and falling by 0.01%. An error correction coefficient of -0.21 indicates that when short-term fluctuations deviate from the long-term equilibrium value, the error term is pulled in the opposite direction with a force of 0.21 to restore equilibrium. In the long and short term, the impact of land sale income on housing prices is slightly greater than is that of infrastructure investment, which further shows that the role of land finance in housing prices in less-developed areas is more obvious than is that in more developed areas. Due to the economic backwardness caused by the location of cities in the central and western regions, the government can increase revenue only by selling land. The level of infrastructure investment is far from sufficient, and the infrastructure itself does not meet the housing environment requirements of home buyers. However, as the central government increases its level of investment in cities in the central and western regions, the economy booms. The continuous improvement in infrastructure and the continuous growth and enrichment of the supply of public services and products strengthen the impact on housing prices.

## 6. Robustness test

### 6.1 Adopting vector error correction model

The lack of control variables in econometric models can lead to spurious regression, because ECM are used for modeling with only two sequences (two-dimensional) and cannot study long-term relationships among multiple variables. Therefore, we use the VECM for investigation, which is used for the relationship test of multiple sequences (multidimensional) and adopts the maximum likelihood estimation method. We add some variables from the supply and demand sides to reanalyze housing prices. The variables selected are as follows: Per capita GDP (pgdp), urbanization rate (urban), regional total population (people), real estate development investment quota (invest), completed area of commercial housing (area), per capita green space (pcgs) and RMB loan balance of financial institutions at the end of the year. All variables are logarithmic to eliminate heteroscedasticity.

The VECM is a kind of VAR with a cointegration constraint, which is often applied to the modeling of nonstationary time series with cointegration relationship. After considering the order of *e*,*k* without exogenous variables is the VAR (*p*).


$$Y_{t}=A_{1}Y_{t-1}+A_{2}Y_{t-2}+\cdots +A_{p}Y_{t-p}+\mu_{t}$$ (3)

In the formula: $\mu_{t}=[\begin{array}{c} \mu_{1t}\\ \mu_{2t}\\ \vdots \\ \mu_{kt} \end{array}]$; $A_{t}=[\begin{array}{cccc} a_{11,j} & a_{12,j} & \cdots & a_{1k,j}\\ a_{21,j} & a_{22,j} & \cdots & a_{2k,j}\\ \vdots & \vdots & & \vdots \\ a_{k1,j} & a_{k2,j} & \cdots & a_{kk,j} \end{array}]$, *j* = 1,2,⋯,3, *p*; and $\mu_{t}=[\begin{array}{c} \mu_{1t}\\ \mu_{2t}\\ \vdots \\ \mu_{kt} \end{array}]$.

Formula (3) is transformed into following:

$$\Delta Y_{t}=\Pi Y_{t-1}+\sum_{i=1}^{p-1}\Pi_{i}\Delta Y_{t-1}+\mu_{t}$$ (4)

In Formula (4), Π = *αβ*′. Matrix *α* is the adjustment coefficient matrix, where each column vector corresponds to a set of weights for each cointegration combination, and matrix *β*′ is the cointegration vector matrix, where each row vector is a cointegration vector. Therefore, Π*Y*_*t*−1_ in Eq (4) can be expressed as *αβ*′*Y*_*t*−1_. Since *β*′*Y*_*t*−1_ is the error-correcting term *ecm*_*t*−1_, Eq (4) can be written as follows:

$$\Delta Y_{t}=\alpha {ecm}_{t-1}+\sum_{i=1}^{p-1}\Pi_{i}\Delta Y_{t-1}+\mu_{t}$$ (5)

The error correction term *ecm*_*t*−1_ reflects the long-term equilibrium relationship among variables, and coefficient matrix *α* reflects the adjustment speed of the error correction term when the equilibrium relationship among variables deviates from the long-term equilibrium state. The coefficient of the difference term of the independent variable reflects the influence of the short-term fluctuation in each variable on the short-term change in the dependent variable.

To reasonably test the impact of various different on housing prices, the specific regression model is as follows:

$${\Delta HP}_{i,t}=\gamma_{1}+\sum_{i=1}^{p-1}\gamma_{2}\Delta {LF}_{it}+\sum_{i=1}^{p-1}\gamma_{3}\Delta {IE}_{it}+\sum_{i=1}^{p-1}\gamma_{4}\Delta {pgdp}_{it}+\sum_{i=1}^{p-1}\gamma_{5}\Delta {urban}_{it}+\sum_{i=1}^{p-1}\gamma_{6}\Delta {people}_{it}+\sum_{i=1}^{p-1}\gamma_{7}\Delta {invest}_{it}+\sum_{i=1}^{p-1}\gamma_{8}\Delta {area}_{it}+\sum_{i=1}^{p-1}\gamma_{9}\Delta {pcgs}_{it}+\sum_{i=1}^{p-1}\gamma_{10}\Delta {loan}_{it}+\sum_{i=1}^{p-1}\gamma_{11}\Delta {HP}_{it-1}-\gamma {ecm}_{it-1}+\varepsilon_{it}$$ (6)

Before the VECM, we perform unit root tests and cointegration tests on all variables. In the unit root test, the p-values of the variables are not significant. None of them can negate the null hypothesis, and the test results are greater than 0.05. Therefore, we performed first-order differentiation on all variables and retest their stationarity, and the p values of the variables are significant at the 1% level, indicating that all variables were stationary and present first-order stationarity after first-order differentiation treatment. In the cointegration test, to ensure the reliability of the conclusion, we use the Kao and Johansen tests, the results as shown in Table 9, that the alternative hypothesis of no cointegration vector and at most 1 cointegration vector are rejected at the significance level of 5%, and thus, the model is considered to have 1 cointegration relationship.

**Table 9:** Cointegration test results.

**Kao’s ADF statistic**				
**Method**	**Hypothesis**	**Statistics**	**t-Statistic**	**P**
Kao	*H*_0_: ρ = 1	ADF	-5.5708	0.0000
**Unrestricted cointegration rank test (maximum eigenvalue)**				
**Hypothesized No. of CE(s)**	**Eigenvalue**	**Max Eigen St.**	**0.05 Critical Value**	**P**
None*	0.2303	33.4305	27.5047	0.0000
At most 1	0.0300	16.0364	21.1316	0.2227

Note: indicates significance at the 5% level.

The Kao test and Johansen cointegration test results show that there is a long-term stable relationship among variables. The maximum eigenvalue test rejects the null hypothesis at a 95% confidence interval and considers that there is one cointegration equation, thus ensuring the feasibility of establishing a VECM. Using the method of maximum likelihood estimation, regression analysis of the VECM is conducted, and the results are shown in Table 10.

**Table 10:** Estimation results of the VECM.

Cointegrating Eq	CointEq1	Error Correction	CointEq1	*C*
HP(-1)	2.8863	*D*(*HP*(−1))	-0.1035(0.0026)	0.1166(0.0191)
LF(-1)	2.7429	*D*(*LF*)	0.0545(0.0126)	0.2283(0.0904)
IE(-1)	-0.8116	*D*(*IE*)	0.0110(0.0043)	0.0964(0.0353)
pgdp(-1)	-3.3737	*D*(*pgdp*)	0.0100(0.0036)	0.0926(0.0260)
people(-1)	-0.9559	*D*(*people*)	-0.0010(0.0005)	0.0129(0.0034)
loan(-1)	-0.6157	*D*(*loan*)	0.0022(0.0029)	0.1828(0.0205)
invest(-1)	-5.1926	*D*(*invest*)	0.0183(0.0024)	0.0858(0.0261)
urban(-1)	-10.1861	*D*(*urban*)	0.0028(0.0008)	0.0224(0.0057)
pcgs(-1)	2.3041	*D*(*pcgs*)	-0.0020(0.0019)	0.0402(0.0139)
area(-1)	1.000	*D*(*area*)	-0.0060(0.0046)	-0.0670(0.0328)
*C*	58.7190			

Note: Standard errors are in parentheses.

The coefficient of the error correction term *ecm*_*t*−1_ indicates that the error correction term has a correcting effect on the housing price, and its effect has a value of -0.1035, indicating that the nonequilibrium error in the previous period reverses the housing price in the current period, with an adjustment force of 0.1035, thus, the housing price return from that in short-term disequilibrium state to that in the long-term equilibrium state. The finding conforms to the reverse correction mechanism and is significantly negative. The coefficient of impact of the level of infrastructure investment on housing prices is 0.011, indicating that an increase of 1% in infrastructure investment increases housing prices by 0.011%. A 1% increase in land sale income leads to a 0.0545% increase in housing prices. The impact of land sale income on housing prices is greater than is that of infrastructure investment. On the one hand, this trend of increasing housing prices has laid the financial foundation for the increase in land transfer prices. On the other hand, the way in which land is sold, supported by the "auction listing" system, has laid the institutional foundation for the increase in land transfer prices. Infrastructure has promoted urban economic development, attracted a great deal of investment, and accelerated the process of urbanization, stimulating people’s housing demand, and thus pushing up housing prices.

### 6.2 Replacing variable representations

In the above analysis, we adopt absolute values to examine the relationship among variables. Due to the heterogeneous characteristics of properties, the average housing price may not reflect such fluctuations. Therefore, we replace the original variables with the growth rates of housing prices, land finance, and infrastructure investment, and perform regression on Eq (1). The results are shown in Table 11. We find significant correlations among the three variables after controlling for year and city fixed effects, which, once again, proves the correlation among housing prices, land finance and infrastructure investment.

**Table 11:** Robustness test of the growth rate.

Variable	HP growth rate	HP growth rate	HP growth rate
LF growth rate	0.163***		0.161***
	(0.0313)		(0.0298)
IE growth rate		0.139***	0.126***
		(0.0535)	(0.0416)
Constant	5.926***	6.649***	5.044***
	(0.319)	(0.370)	(0.331)
City	Control	Control	Control
Year	Control	Control	Control

## 7. Conclusions and policy implications

This paper empirically demonstrates the impact of land transfer income and infrastructure investment on housing prices from the perspective of government revenue and expenditure. Based on the relevant data of 35 large and medium-sized cities in China from 2000 to 2017, the conclusions drawn in this paper are presented below.

First, under Granger’s causality test, we find that real estate prices, land transfer income and infrastructure investment have causal effects on one another. In addition, we find that under the pressure of officials’ promotion, local governments’ infrastructure investment is overly reliant on land finance. Second, through the ECM, the long-term elasticity of land transfer income and infrastructure investment to housing prices is much greater than the short-term elasticity. It can be inferred that the effect of land finance and infrastructure investment on housing prices has significantly increased over time. Third, the results for the eastern, central and western regions show that the drivers of land finance are more pronounced in less-developed regions than in more developed regions.

The above findings put forwards some possible policy implications for China’s housing market regulation. First, the high reliance of local governments on land finance should be gradually abandoned. Local governments should implement counter-cyclical regulation in accordance with the real estate market environment, optimize the conditions of land transfers and prevent unreasonable increases in land prices. Second, we suggest that the central government support diversified financing for local governments, especially by increasing credit support for infrastructure investment by local governments. Local governments should pay attention to changing the mode of revenue and expenditure, and implement policies according to the city’s local conditions. Third, we recommend that the central government narrow the degree of regional disparities. It is necessary to strengthen the supervision of the land market in the eastern region and balance the fiscal expenditure among regions. Thus, a land supply management mechanism should be established in the central and western regions, and a rational local fiscal revenue and expenditure plan should be formulated.

Although this study investigates the relationship between housing prices, land finance and infrastructure investment, some limitations of this study deserve further exploration. First, the house price index could have been a better dependent variable in reflecting the price change based on supply and demand forces. However, the official source of the annual city-level housing price index is unavailable at present. We have to adopt the average housing prices to keep in line with the absolute values of land financing and infrastructure investment. Second, the data of land transfer income is only available until 2017. How to comprehensively analyze the current state of the real estate market is worthy of further study.
